# Efficacy and safety of add-on anti-CD20 monoclonal antibody to Bruton tyrosine kinase inhibitor treatment for chronic lymphocytic leukemia: a meta-analysis

**DOI:** 10.1038/s41598-023-36279-x

**Published:** 2023-06-16

**Authors:** Thi Thuy Nguyen, Nguyen Thanh Nhu, Van Khoi Tran, Nguyen-Kieu Viet-Nhi, Xuan Dung Ho, Ming-Kai Jhan, Ya-Ping Chen, Chiou-Feng Lin

**Affiliations:** 1 International Ph.D. Program in Medicine, College of Medicine, Taipei Medical University, Taipei, 110 Taiwan; 2 Department of Oncology, Hue University of Medicine and Pharmacy, Hue University, Hue, Vietnam; 3 Faculty of Medicine, Can Tho University of Medicine and Pharmacy, Can Tho, Vietnam; 4 Department of Surgery, Hue University of Medicine and Pharmacy, Hue University, Hue, Vietnam; 5 Department of Microbiology and Immunology, School of Medicine, College of Medicine, Taipei Medical University, No. 25, Wuxing St, Xinyi District, Taipei, 110 Taiwan; 6 Graduate Institute of Medical Sciences, College of Medicine, Taipei Medical University, Taipei, 110 Taiwan; 7 Division of Hematology, Department of Internal Medicine, National Cheng Kung University Hospital, College of Medicine, National Cheng Kung University, Tainan, 704 Taiwan; 8 Core Laboratory of Immune Monitoring, Office of Research & Development, Taipei Medical University, Taipei, 110 Taiwan

**Keywords:** Targeted therapies, Chronic lymphocytic leukaemia

## Abstract

The efficacy of Bruton tyrosine kinase inhibitors (BTKi) remains suboptimal in chronic lymphocytic leukemia (CLL) treatment. A systematic review and meta-analysis were conducted to compare the outcomes of combining anti-CD20 monoclonal antibodies (mAb) with BTKi therapy versus BTKi monotherapy for patients with CLL. We searched for relevant studies in the Pubmed, Medline, Embase, and Cochrane databases until December 2022. We estimated the effective results using a hazard ratio (HR) for survival outcomes and relative risk (RR) for response outcomes and safety. Four randomized controlled trials (including 1056 patients) were found until November 2022 and fulfilled the inclusion criteria. Progression-free survival was significantly improved with the addition of anti-CD20 mAb to BTKi over BTKi (HR 0.70, 95% confidence interval (CI) 0.51–0.97), whereas pooled analysis of overall survival did not favor combination therapy compared to BTKi monotherapy (HR 0.72, 95% CI 0.50–1.04). Combination therapy was related to a statistically better complete response (RR, 2.03; 95% CI 1.01 to 4.06) and an undetectable minimal residual disease rate (RR, 6.43; 95% CI 3.54 to 11.67). The risk of grade ≥ 3 adverse events was comparable between the two groups (RR, 1.08; (95% CI 0.80 to 1.45). Overall, adding anti-CD20 mAb to BTKi revealed superior efficacy than BTKi alone in untreated or previously treated CLL patients without affecting the safety of single-agent BTKi. Conducting further randomized studies to confirm our results and determine the optimal therapy for managing patients with CLL is essential.

## Introduction

Chronic lymphocytic leukemia (CLL) is an indolent B-cell malignancy characterized by the accumulation of mature-looking CD19+ CD23+ CD5+ B cells within the bone marrow, peripheral blood, and lymphoid organs^1^. Similar to most cancers, CLL is a heterogeneous disease with various known genetic alterations, such as 17p deletion (del[17p]), tumor protein 53 (TP53) mutation, and 11q deletion (del[11q]), which have been identified as unfavorable prognostic markers in patients treated with chemoimmunotherapy^2,3^. Among patients ≥ 65 years old, nonchemotherapeutic drugs targeting the signaling pathway, such as B-cell receptor (BCR) signaling inhibitors or the BCL-2 antagonist venetoclax, have shown superior outcomes compared with chemoimmunotherapy as the standard treatment^4,5^. However, combination chemotherapy with anti-CD20 monoclonal antibodies (mAb) has greatly improved efficacy^6–8^. Despite progress in the advanced treatment of CLL, some patients relapse or become refractory upon repeated chemoimmunotherapy^9^. Of them, at least 30% of patients with high-risk genomic features could relapse after achieving a response to first-line treatment^10^. Bruton tyrosine kinase inhibitors (BTKi) (Ibrutinib, Acalabrutinib, and Zanubrutinib), which block the BCR signaling cascade by binding to BTK, are selective irreversible inhibitors and approved by the Food and Drug Administration and the European Medicine Agency for the treatment of patients with untreated, relapsed, or refractory disease^11–14^. Several previous randomized controlled trials (RCTs)^5,11,15^ have shown better progression-free survival (PFS) outcomes of BTKi with or without anti-CD20 mAb compared with chemoimmunotherapy in CLL treatment. However, it remains unclear whether adding of anti-CD20 mAb to BTKi provides greater efficacy than BTKi monotherapy. Previous extensive studies have reported that BTKi plus anti-CD20 mAb therapy did not enhance the likelihood of PFS versus BTKi monotherapy in CLL patients^5,16^. In contrast, other recent studies have shown the superior efficacy of BTKi in combination with next-generation anti-CD20 mAb compared with BTKi alone among CLL patients^17,18^. The possible addition of anti-CD20 mAb therapy to BTKi leads to improved outcomes for CLL patients is controversial. In addition, no published systematic review and meta-analysis has been conducted to compare the efficacy of BTKi as monotherapy or in combination with anti-CD20 mAb in treatment-naïve or relapsed/refractory CLL patients, including those with high-risk cytogenetics CLL. Hence, this study aimed to provide all evidence and compare the clinical effectiveness and safety of BTKi plus anti-CD20 mAb versus BTKi monotherapy for CLL.

## Methods

### Registration and protocol

The review protocol was registered on the “International Prospective Register of Systematic Review” under CRD42022368514. In addition, the review was performed as reported by the “Preferred Reporting Items for Systematic Reviews and Meta-Analyses” (PRISMA) 2020 guidelines^19^.

### Data sources and search strategies

We searched PubMed, Embase, Medline, and Cochrane Library databases until November 2022 to retrieve relevant articles without the limitations of type, country, or language (detailed search strategy provided in Table S1). We also scanned the reference lists of the included studies to search for other relevant publications. Two researchers independently performed the search procedure. If disagreement occurred, another researcher was consulted. A final decision was made after all researchers could unequivocally resolve discrepancies with full agreement.

### Study selection

We included all RCTs comparing BTKi (including ibrutinib, acalabrutinib, zanubrutinib, etc.) plus anti-CD20 mAb (including Rituximab, Ublituximab, Obinutuzumab, etc.) versus BTKi monotherapy for untreated and/or relapsed/refractory CLL. All trials were selected without restrictions on place or country, study quality, or follow-up time. Titles and abstracts were reviewed to weed out duplicates and irrelevant publications that lacked the requisite information on Endnote X9 (Clarivate. Philadelphia, PA, USA). The full text of each study was read, and those that met the qualifying requirements were included. Two independent evaluators conducted the selection procedure. Two authors conferred with a third consultant when a dispute arose.

### Data extraction and quality assessment

Two independent authors performed the data extraction process. First, the included papers’ main text, tables, and graphs were read to extract information, including RCT characteristics and outcomes. If data were unavailable, we requested them from the corresponding authors. The ultimate decision was made after discussing the differences with a third reviewer about the disagreement between the two reviewers. For data extraction, the following data were extracted: first author, year of publication, country, study design, accrual period, ClinicalTrials.gov Identifier, sample size, length of median follow-up, patient characteristics, Rai staging, immunoglobulin heavy chain gene (IGHV) status, genetic abnormalities consisting of del[11q], del[17p] and TP53, unmethylated encoding zeta chain–associated protein kinase (ZAP) 70, treatment parameters, primary outcomes, and different outcomes. If multiple records were published with different follow-up times and referred to the same data, the results from the most recent publication were cross-checked against the previous publication, and the most recent data were included in the final analysis.

Using the criteria specified in the “Cochrane Handbook for Systematic Reviews of Interventions version 6.3”, the methodological quality and risk of bias of the included RCTs were assessed^20^. Two researchers separately assessed the trials, cross-checked, and filled out the predesigned datasheets for the Cochrane risk-of-bias tool for randomized trials (ROB 2) checklists. The allocation concealment, generation of the allocation bias, blinding of participants and investigators, blinding of outcome measurement, incompletely reported outcomes, and selective outcome reporting were checked carefully. Finally, another researcher resolved the disagreement.

### Outcome evaluation

Primary outcomes were both PFS and overall survival (OS). Secondary outcomes were the overall response rate (ORR), complete response (CR), partial response (PR), undetectable minimal residual disease (uMRD) rate, and safety. PFS was defined as the time from the randomization date to disease progression or death from any cause. OS was defined as the date from random assignment until death due to any cause. We defined the ORR as the sum of CR, CR with incomplete bone marrow recovery, PR, nodular PR, or PR with lymphocytosis. The CR rate includes CR and CR with incomplete bone marrow recovery. The PR rate was evaluated based on the proportion of patients with PR, nodular PR, or PR with lymphocytosis. An independent review committee conducted response evaluation in four trials following the 2008 International Workshop on Chronic Lymphocytic Leukaemia (IWCLL) criteria^1^. Adverse events (AEs) were also performed by “the National Cancer Institute Common Terminology Criteria for Adverse Events, version 4.03”. MRD assessment was conducted using flow cytometry with a cut-off of 10^–4^ for uMRD. uMRD rates were defined as the number of cases with negative MRD patients out of the total number of randomized patients.

### Data synthesis and statistical analysis

Time-to-event outcomes (PFS, OS) were calculated and pooled to hazard ratios (HRs) in R software (R Foundation for Statistical Computing; Vienna, Austria). Treatment effect (TE) and standard errors of TE (seTE) determined from 95% confidence intervals (CI) estimating a normal distribution in a log-transformed scale was utilized in the analysis of HR. An HR of < 1.0 was in favor of the combination treatment. If HRs were not available in a trial, but the corresponding Kaplan‒Meier curves were reported, we used the algorithm Tierney et al.^21^ described to reconstruct the HR from digitized curves, combined with the patients at risk and the sum of events. Relative risk (RRs) and 95% CIs for binary outcomes were calculated and pooled using the standard Mantel–Haenszel method. Pooled effect sizes of each outcome were computed using a random effect model. Subgroup analyses were calculated to explore the sources of heterogeneity among studies. Hedges Q and I^2^ statistics were used to judge and quantify the magnitude of heterogeneity, with I^2^ higher than 50% being considered significant heterogeneity.

## Results

### Study characteristics

**Figure 1:**
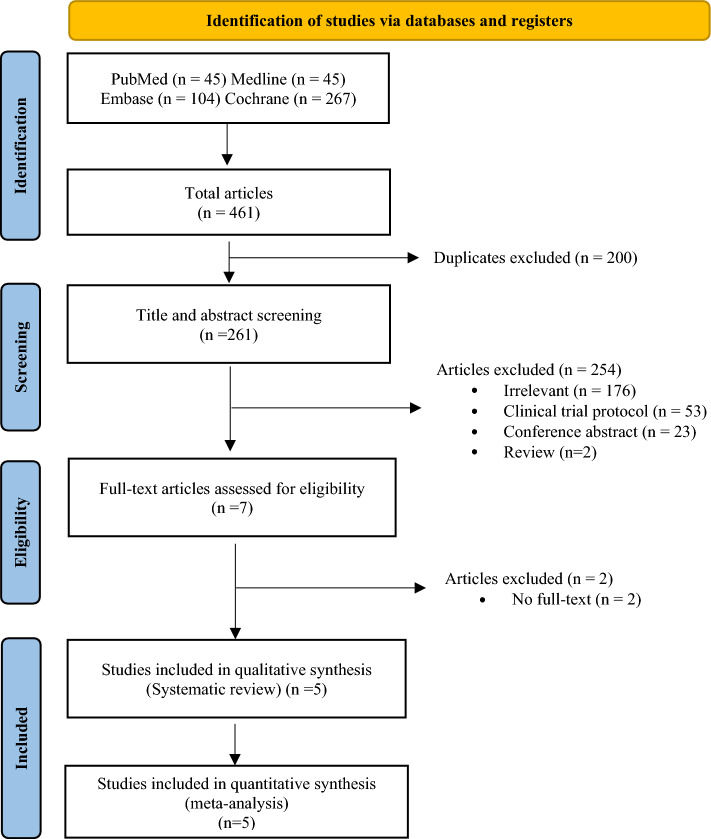
PRISMA flowchart summarizing the study selection process.

A total of 461 publications were obtained from the electronic databases through a systematic search, of which 261 were considered relevant. Overall, 254 were excluded for several reasons (Fig. 1). Five publications remained for the eligibility assessment^5,16–18,22^ but only four trials met the inclusion criteria in the final analysis because two publications with long-term follow-up published over time were determined^17,22^. RCTs were published from 2018 to 2021 and were conducted in multiple countries between 2013 and 2017 (Table 1).

**Table 1:** Characteristics of the included trials.

	NR Burger et al. (2019)	GENUINE Sharman et al. (2021)	ELEVATE-TN Sharman et al. (2020)	ALLIANCE Woyach et al. (2018)
Study design	Phase 2, SC, OL	Phase 3, MC, OL	Phase 3, MC, OL	Phase 3, MC, OL
Enrolled period	12/2013–10/2017	02/2015–12/2016	09/2015–02/2017	12/2013–05/2016
ClinicalTrials.gov ID	NCT02007044	NCT02301156	NCT02475681	NCT01886872
Method of analysis	ITT	ITT	ITT	ITT
CLL definition	iwCLL 2008 criteria	iwCLL 2008 criteria	iwCLL 2008 criteria	iwCLL 2008 criteria
Response definition	iwCLL 2008 criteria	iwCLL 2008 criteria	iwCLL 2008 criteria	iwCLL 2008 criteria
Safety assessment	CTCAE v4.03	CTCAE v4.03	CTCAE v4.03	NR
Inclusion criteria	Relapsed and treatment-naive high-risk patients with CLL	Relapsed or refractory high-risk patients with CLL	Treatment-naive patients with CLL	Treatment-naive patients with CLL
Randomization	1:1	1:1	1:1	1:1
Masking	No	No	No	No
Randomization stratification	High-risk cytogenetic abnormalities (TP53 mutation/del17p, del11q without del17p or TP53 mutation, or none/unknown) and ECOG PS (0–1 vs. 2)	Previous therapy (one vs. two or more regimens) with a block size of four	Presence or absence of del (17) (p13·1), ECOG PS score (0–1 vs. 2), and geographic region	Risk factors for CLL
Primary outcome	PFS	ORR	PFS	PFS
Secondary outcome	ORR	PFS	ORR	OS
Definition PFS	The number of months from the treatment date to the progression or death date	The interval from randomization until disease progression or death from any cause	The time from randomization until disease progression or death	The time from the date of randomization until the earliest date on which disease progression or death from any cause was recorded
Intervention arm	Ibrutinib 420 mg/day + Rituximab (375 mg/m^2^)	Ibrutinib 420 mg/day + Ublituximab (900 mg)	Acalabrutinib 200 mg/day + Obinutuzumab (1000 mg)	Ibrutinib 420 mg/day + Rituximab (375 mg/m^2^)
Control arm	Ibrutinib 420 mg/day	Ibrutinib 420 mg/day	Acalabrutinib 200 mg/day	Ibrutinib 420 mg/day
Sample size	208	126	358	364
Time of initial response assessment	After 3 or 6 cycles	After completion of cycles 2, 4, 6	Week 12	NR

*NR*, not reported; *SC*, single-center; *MC*, multicenter; *OL*, open-label; *ID*, identifier; *ITT*, intention-to-treat; *CLL*, Chronic Lymphocytic Leukaemia; *iwCLL*, International Workshop on Chronic Lymphocytic Leukaemia; *ECOG PS,* Eastern Cooperative Oncology Group performance status; *CTCAE*, the US National Cancer Institute Common Terminology Criteria for Adverse Events; *PFS*, progression-free survival; *ORR*, overall response rate; *OS*, overall survival.

### Description of patients

Our study included 1056 patients (Table 2). The diagnosis of CLL in all four RCTs was in accordance with the 2008 IWCLL criteria^1^. Patients were between 42 and 89 years old, with males accounting for 67%. The median follow-up time ranged from 0 to 59.4 months. According to the investigator's evaluation, all cases had to be eligible for BTKi or anti-CD20 mAb treatment. All the analyzed studies recruited either treatment-naïve or relapsed/refractory patients with CLL. While two studies (ELEVATE-TN trial and ALLIANCE trial) were conducted in previously untreated patients, the other two studies (GENUINE trial and Burger et al. trial) were primarily performed in the relapsed/refractory setting. The high-risk cytogenetic abnormalities were assessed at a similar rate in all trials, except for unmethylated ZAP70 reported in two trials^5,16^.

**Table 2:** Characteristics of patients included in the trials.

Study (first author, year)	BTK inhibitor + anti-CD20 arm/ BTK inhibitor arm	Patient, n	Patient age, median (range)	Sex, male, n (%)	ECOG PS (0–1), n (%)	ECOG PS (2), n (%)	Rai stage (III–IV), n (%)	Treatment-naïve patients, n (%)	Relapsed or refractory patients, n (%)	Number of patients with IGHV unmutated, n (%)	Number of patients with TP53 mutated, n (%)	Number of patients with del17p, n (%)	Number of patients with 11q deletion, n (%)	Number of patients with complex karyotype	Number of patients with unmethylated ZAP70	Follow-up, month, median (range)
Burger et al. (2019)	Ibrutinib + Rituximab	104	65 (42–81)	71 (68)	104 (100)	0 (0)	42 (40)	12 (12)	92 (88)	62 (60)	21 (20)	30 (29)	15 (14)	NR	63 (61)	36.4 (2.9–47.8)
	Ibrutinib	104	65 (42–81)	75 (72.1)	104 (100)	0 (0)	38 (37)	15 (14)	89 (86)	61 (59)	29 (28)	26 (25)	27 (26)		65 (63)	35.8 (3.0–47.1)
Sharman et al. (2021)	Ibrutinib + Ublituximab	64	66 (62–74)	44 (69)	63 (98)	1 (2)	31/61 (51)	0 (0)	64 (100)	53 (83)	20 (31)	28 (44)	30 (47)	NR	NR	41.6 (36.7–47.3)
	Ibrutinib	62	67 (62–74)	46 (74)	60 (97)	2 (3)	26/59 (44)	0 (0)	62 (100)	52 (84)	28 (45)	30 (48)	27 (44)			
Sharman et al. (2020)	Acalabrutinib + Obinutuzumab	179	70 (65–75)	111 (62)	169 (94.4)	10 (5.6)	86 (48.0)	179 (100)	179 (100)	103 (57.5)	21 (11.7)	17 (9.5)	31 (17.3)	29 (16.2)	NR	46.9 (0.0–59.4)
	Acalabrutinib	179	70 (66–75)	111 (62)	165 (92.2)	14 (7.8)	87 (48.6)	179 (100)	0 (0)	119 (66.5)	19 (10.6)	16 (8.9)	31 (17.3)	31 (17.3)		
Woyach et al. (2018)	Ibrutinib + Rituximab	182	71 (65–86)	125 (69)	180 (99)	2 (1)	98 (54)	182 (100)	0 (0)	70/115 (61)	20/168 (12)	11/180 (6)	37/180 (21)	60/168 (36)	96/182 (53)	38
	Ibrutinib	182	71 (65–89)	123 (68)	177 (97)	5 (3)	99 (54)	182 (100)	0 (0)	77/122 (63)	15/168 (9)	9/181 (5)	35/181 (19)	39/165 (24)	96/182 (53)	

*BTK* Bruton tyrosine kinase, *ECOG PS* Eastern Cooperative Oncology Group performance status, *IGHV* unmutated immunoglobulin heavy chain variable, *ZAP70* Encoding zeta chain-associated protein kinase 70 *NR* not reported.

### BTKi and anti-CD20 mAb procedure

After enrolling, patients were randomly assigned (1:1) to receive BTKi plus anti-CD20 mAb or single-agent BTKi. Treatments were administered in 28-day cycles. Patients in the BTKi site received ibrutinib 420 mg per oral dose daily (1 cycle = 28 days)^5,16,18^, while in the ELEVATE-TN trial^17,22^, oral acalabrutinib was taken (100 mg) twice a day. Ibrutinib or acalabrutinib was given until progressive disease, unacceptable toxic effects, or consent withdrawal of trials. At the anti-CD20 mAb site, rituximab was administered 375 mg/m^2^ intravenously weekly for weeks 1 to 4 (on cycle 1^16^ or cycle 2^5^) and was administered once every 4 weeks until cycle 6. In the GENUINE trial^18^, intravenous Ublituximab was given on Days 1 (≤ 150 mg), 2 (750 mg), 8 (900 mg), and 15 (900 mg) of cycle 1 and on Day 1 (900 mg) of cycles 2–6. Patients who received Ublituximab (900 mg) remained on treatment every three cycles after cycle 6 until they had unacceptable toxicity or disease progression. In the ELEVATE-TN trial, obinutuzumab was administered intravenously on Day 1 (100 mg), Day 2 (900 mg), Days 8 and 15 (1000 mg) of cycle 2, and Day 1 of cycles 3–7 (1000 mg). Study drugs could be delayed in the event of significant treatment-related toxicity; however, no reduction in the anti-CD20 mAb dose was allowed.

### Risk of bias

All four RCTs were considered to have some bias concerns due to deviations from the intended intervention (Table 3). Although these trials were reported as open-label, a masked independent review committee evaluated response data and disease progression^17,18^.

**Table 3:**
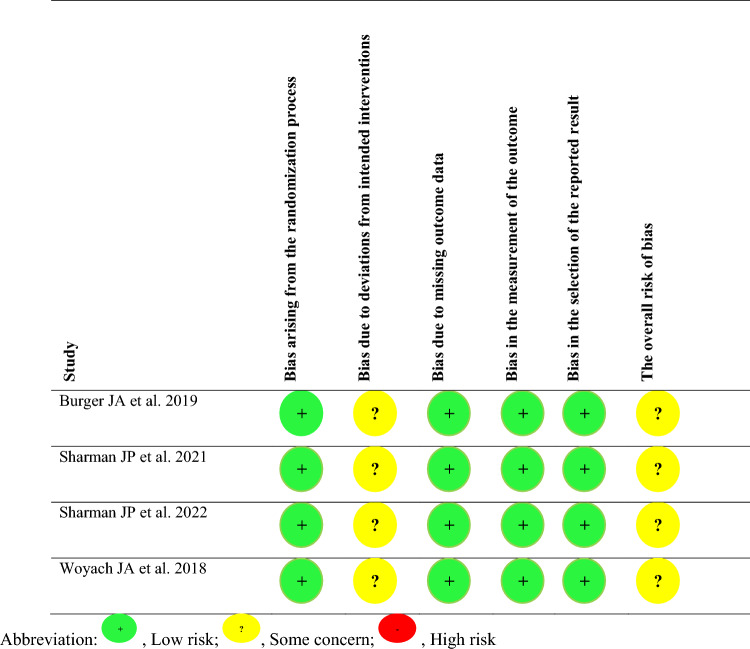
Risk of bias evaluation of the randomized controlled trials using the ROB 2 tool.

### Primary endpoints

**Figure 2:**
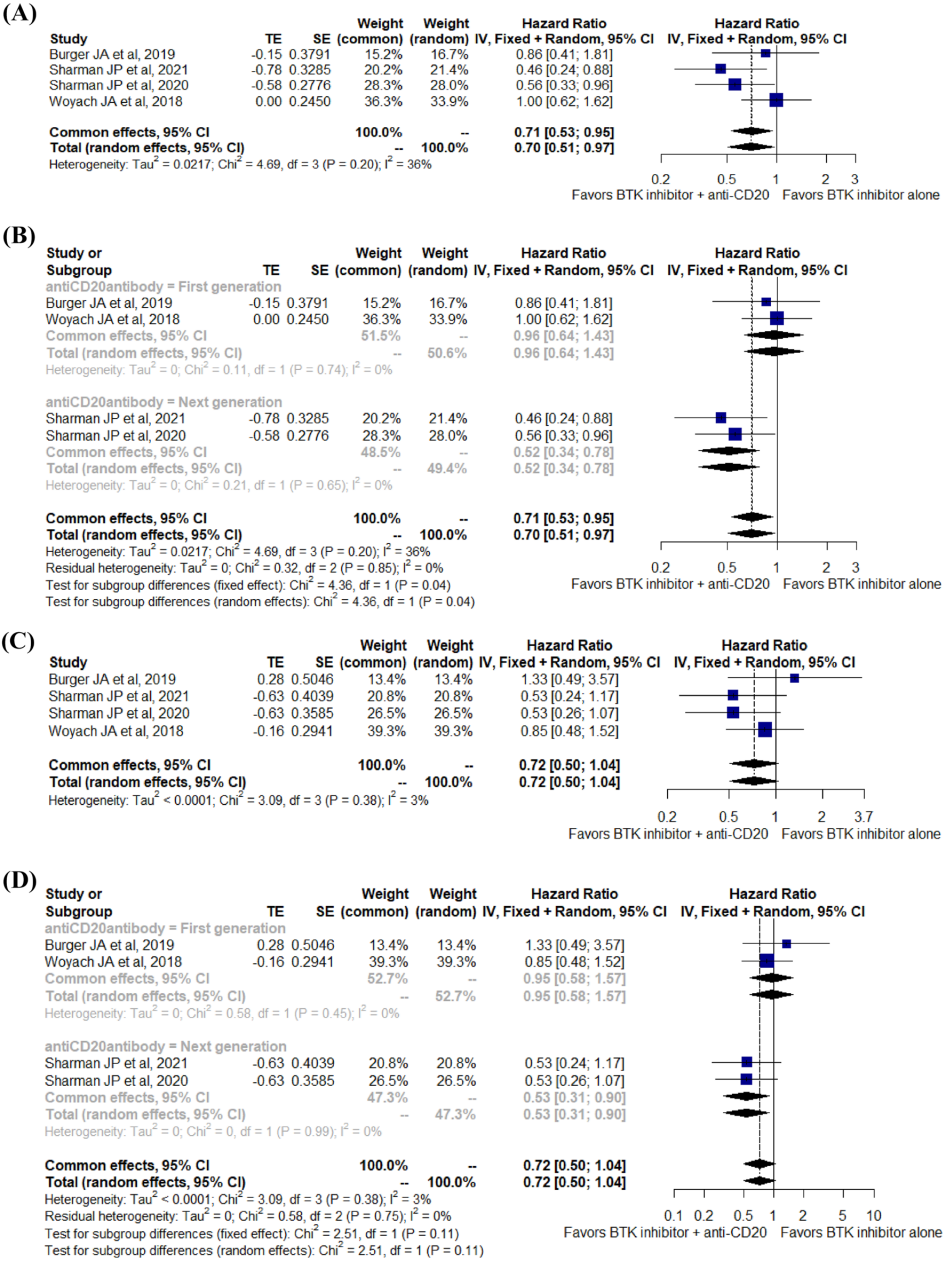
Forest plot for progression-free survival (**A**), subgroup analysis of progression-free survival (**B**), overall survival (**C**), and subgroup analysis of overall survival (**D**).

Data from all RCTs were reported for analysis of PFS. Of note, OS data were available for analysis except for the ALLIANCE trial. Hence, the algorithm described by Tierney et al*.*^21^ was used to acquire an estimate of the HR and 95% CI from the corresponding Kaplan‒Meier curves reported in the ALLIANCE study. PFS was significantly improved with BTKi plus anti-CD20 mAb therapy compared to BTKi monotherapy (HR 0.70; 95% CI 0.51–0.97; I^2^ = 36%; p = 0.20; 1031 patients; four RCTs) (Fig. 2A). A subgroup analysis between next generation anti-CD20 antibodies and fist generation anti-CD20 antibody was conducted to see if endpoints yield more significant benefit with the exclusion of the two rituximab trials. Among patients receiving obinutuzumab/ublituximab, the PFS advantage was also statistically significant (HR 0.52; 95% CI 0.34–0.78; I^2^ = 0%; p = 0.65; 475 patients; two RCTs) (Fig. 2B). However, the OS advantage was not statistically significant (HR 0.72; 95% CI 0.50–1.04; I^2^ = 3%; p = 0.38; 1031 patients; four RCTs) (Fig. 2C). An additional analysis was performed by combining the two studies with next generation anti-CD20 antibodies to check the benefits of obinutuzumab/ublituximab. Subgroup analysis of OS among patients receiving next generation anti-CD20 antibodies favored the combination therapy over the BTKi monotherapy (HR 0.53; 95% CI 0.31–0.90; I^2^ = 0%; p = 0.99; 475 patients; two RCTs) (Fig. 2D). However, among del[17p] and/or mutated TP53 patients, PFS was not significantly improved with the combination of BTKi and anti-CD20 mAb therapy when compared with BTKi alone (HR 0.63; 95% CI 0.32–1.24; I^2^ = 44%; p = 0.15; 193 patients; four RCTs). Subgroup analysis of PFS with patients del[11q] also did not favor the addition of an anti-CD20 agent to BTKi over BTKi alone (HR 1.20; 95% CI 0.60–2.40; I^2^ = 0%; p = 0.84; 186 patients; three RCTs) (Fig. S1A,B).

### Secondary endpoints

**Figure 3:**
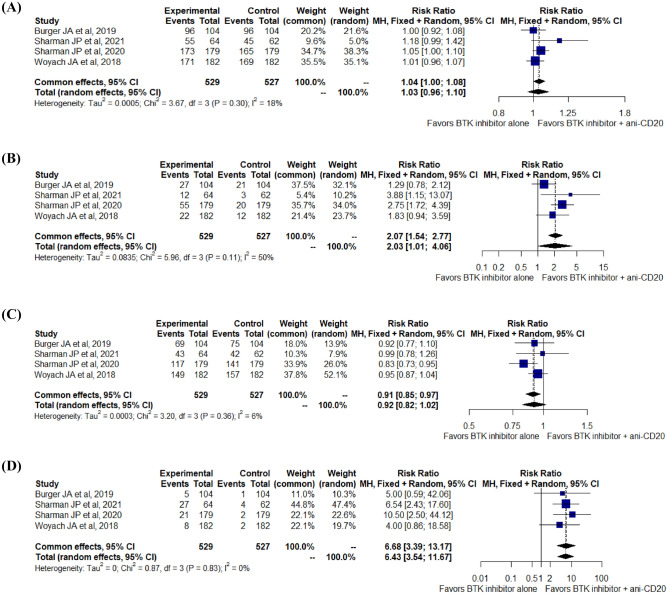
Pooled relative risk for overall response (**A**), complete response (**B**), partial response (**C**), undetectable minimal residual disease rate (**D**) to treatment.

Data from four RCTs did not show an improved ORR (RR, 1.03; 95% CI 0.96–1.10; I^2^ = 18%; p = 0.30; 1056 patients; four RCTs) or PR rate (RR, 0.92; 95% CI 0.82–1.02; I^2^ = 6%; p = 0.36; 1056 patients; four RCTs) in the BTKi group compared with the BTKi group. However, combining BTKi with anti-CD20 mAb therapy was associated with a significantly better CR rate (RR, 2.03; 95% CI 1.01–4.06; I^2^ = 50%; p = 0.11; 1056 patients; four RCTs) and uMRD rate (RR, 6.43; 95% CI 3.54–11.67; I^2^ = 0%; p = 0.83; 1056 patients; four RCTs) (Fig. 3). A subgroup analysis was performed by combining the two trials with obinutuzumab/ublituximab and the two rituximab trials (Fig. S2A,B).

### Safety

The data showed no difference in the risk of grade AEs between the two arms (RR, 1.00; 95% CI 0.99–1.01; I^2^ = 0%; p = 0.90; 682 patients; three RCTs). Information from all RCTs was available to analyze grades ≥ 3 AEs of particular interest. Similarly, the risk of grade 3 or higher AEs reported was comparable between the two groups (RR, 1.08; 95% CI 0.80–1.45; I^2^ = 77%; p ≤ 0.01; 1043 patients; four RCTs). Regarding haematologic AEs, the risk of grade 3 or higher neutropenia was significantly increased in the combined BTK inhibitor with anti-CD20 treatment arm versus the BTK inhibition treatment arm, 119/522 (23%) versus 64/521 (12%) (RR, 1.80; 95% CI 1.03–3.17; I^2^ = 37%; p = 0.19; 1043 patients; four RCTs). Additionally, there was an increased risk of grade ≥ 3 secondary primary malignancies among the combination treatments, 64/522 (12%) in the anti-CD20 mAb plus BTKi arm versus 55/521 (10%) in the BTKi monotherapy arm (RR, 1.16; 95% CI 1.04–1.30; I^2^ = 0%; p = 0.99; 1043 patients, four RCTs). Regarding other specific grades ≥ 3 AEs, no statistically significant differences were observed between patients with BTKi + /- anti-CD20 antibodies (Table 4). The two groups had no difference in the risk of discontinuing treatment and death from any cause between the two groups (Fig. S2A,B). All-grade (11%) or grade ≥ 3 (1.3%) infusion-related reactions were more frequent with the combination therapy than with BTKi alone (Table S2).

**Table 4:** Summary of pooled relative risk for grade 3 or higher adverse events.

Adverse events	BTK inhibitor + anti-CD20		BTK inhibitor alone		Risk ratio	95% Confident interval	I^2^ (%)	P value
	Events	Total	Event	Total				
AE any grade	334	341	332	341	1.00	0.99–1.01	0	0.90
AE of grade 3 or higher	373	522	336	521	1.08	0.80–1.45	77	< 0.01
Anemia	28	522	38	521	0.74	0.41–1.34	0	0.62
Arthralgia	6	522	5	521	1.15	0.14–9.68	0	0.55
Atrial fibrillation	22	522	28	521	0.79	0.24–2.56	15	0.31
Cough	2	522	5	521	0.55	0.04–8.43	0	0.56
Diarrhea	22	522	13	521	1.58	0.22–11.56	43	0.15
Fatigue	14	522	18	521	0.86	0.27–2.74	0	0.4
Febrile neutropenia	15	522	8	521	1.83	0.34–9.76	13	0.33
Headache	6	522	10	521	0.60	0.34–1.07	0	0.95
Hemorrhage	16	522	17	521	0.94	0.61–1.45	0	0.92
Hypertension	103	522	93	521	1.10	1.00–1.22	0	0.98
Infection	95	522	89	521	1.04	0.43–2.51	53	0.12
Nausea	2	522	4	521	0.65	0.03–15.9	0	0.44
Neutropenia	119	522	64	521	1.80	1.03–3.17	37	0.19
Pneumonia	34	522	24	521	1.38	0.75–2.57	0	0.65
Sepsis	17	522	8	521	1.97	0.96–4.07	0	0.83
SPM	64	522	55	521	1.16	1.04–1.30	0	0.99
Thrombocytopenia	32	522	23	521	1.32	0.39–4.49	39	0.18
URTI	6	522	5	521	0.91	0.08–10.04	15	0.32
UTI	7	522	9	521	0.75	0.08–6.74	0	0.41

*AE* adverse event, *BTK* Bruton tyrosine kinase, *SPM* secondary primary malignancies, *URTI* upper respiratory tract infection, *UTI* Urinary tract infection.

## Discussion

Our study demonstrates a significantly better outcome by adding anti-CD20 mAb to BTKi therapy in untreated or relapsed/refractory patients with CLL. The combined anti-CD20 antibody with BTKi was related to improved PFS, CR rate, and MRD negativity rate, with acceptable tolerability. However, all RCTs showed that adding anti-CD20 mAb to the BTKi was not associated with significant improvements in OS, ORR, or PR. Except for infusion-related reactions associated with anti-CD20 antibody and slightly increased risk of grade ≥ 3 neutropenia and secondary primary malignancies, the combination therapy did not significantly change the safety profile of BTKi.

All four RCTs were innovative in the treatment landscape for CLL. They provided the first effort to challenge the advantage of adding anti-CD20 mAb to BTKi in patients with CLL. Previously, the addition of anti-CD20 antibodies to chemotherapy was associated with significantly improved outcomes^8,23^, and the role of novel anti-CD20 mAbs in combinations, such as bendamustine^24^, PI3K inhibitor^25^, high-dose corticosteroid^26^, and lenalidomide^27^, has been evaluated among patients with relapsed/refractory CLL. Although previous experience with ibrutinib plus rituximab showed high response rates and safety, none has clarified the potential benefit of adding anti-CD20 mAb therapy with BTKi. In the phase 3 iLLUMINATE study^11,28^, the addition of obinutuzumab to BTKi showed superior efficacy compared with chlorambucil plus obinutuzumab (a standard treatment highly recommended in clinical practice guidelines^29^) in first-line treatment of CLL, including in patients with high-risk disease features. With similar follow-up duration in our included trials, this study suggests that the add-on obinutuzumab to BTKi improves the percentage of patients achieving a response, particularly in patients with high-risk CLL. Once again, our meta-analysis confirms adding anti-CD20 therapy (particularly next generation antibodies) to BTKi. Burger et al. showed that the combination of rituximab and BTKi demonstrated quicker and deeper response than BTKi monotherapy, but the PFS with combination therapy was similar to single-agent BTKi^16^. Next generation mAbs used in these studies^11,17,18^ can be attributed to an essential factor in the combination therapy between BTKi and anti-CD20 such as obinutuzumab, which has shown superiority over rituximab in the CLL11 trial^8^. In addition, overall long-term safety profiles in the combination therapy were consistent with the known safety signals of BTKi monotherapy. Neutropenia and infusion-related reactions (IRRs) were more familiar with combination treatment than single-agent BTKi, consistent with a previous study of BTKi combined with anti-CD20 mAb therapies^30^. Indeed, IRRs are potentially serious limitations in the administration of mAbs. When compared with rituximab, the obinutuzumab infusion is significantly associated with more frequent and more severe IRRs as shown in the CLL11 study^8^. Combined with BTKi therapy, IRRs of any grade or grade ≥ 3 were much less common in the combination therapy arm than in chemoimmunotherapy. The mechanism underlying the reduced risk of obinutuzumab-associated IRR in combination with ibrutinib remains unknown, but Greil et al. showed that ibrutinib pretreatment decreases cytokine and chemokine release and reduces the incidence of obinutuzumab-induced IRRs in patients with CLL^31^. This could provide significant clinical relevance in patients treated with mAb therapy and warn clinicians in the combination treatment between Btki and anti-CD20 mAbs.

A preclinical trial combining anti-CD20 mAbs and ibrutinib in CLL indicated that ibrutinib mediates positive and negative interactions on anti-CD20 mAb activities^32^. On the one hand, previous preclinical studies demonstrate multiple negative interactions between BTKi and anti-CD20 antibodies in B-cell malignancies^33–36^. BTKi significantly reduced CD20 expression on CLL cells in vitro and in vivo^32,33^, subsequently decreased anti-CD20 mAb-mediated complement-dependent cytotoxicity (CDC)^32,36^, and diminished antibody-dependent cellular cytotoxicity (ADCC) by directly inhibiting Fc receptor–stimulated NK cell activation and cytotoxicity in vitro in the BTKi and CD20-targeting antibody combination treatment^34^. Similarly, BTKis significantly inhibit antibody-dependent phagocytosis of CLL cells by polymorphonuclear neutrophils and macrophages^36^. The investigation on the impact of BTKi effects on the biological activity of rituximab versus next generation anti-CD20 antibodies is lacking. On the other hand, BTKi potently reduces trogocytosis, a significant mechanism of antigen loss and tumor escape from the combination therapy^32^. Additionally, the Fc portion of the antibody is an important component of Fc-receptor-induced phagocytosis on macrophages and Fc-receptor dependent CD20 loss through trogocytosis^37–39^. The afucosylated Fc portion appears to differ between rituximab and next generation ani-CD20 mAbs^40–42^. Therefore, the positive and negative interaction between BTKi-rituximab and BTKi-Fc-optimized anti-CD20 mAbs could not have the same effect. However, no preclinical and clinical studies have been conducted to compare these interactions, suggesting that such combination therapies need further investigation in vitro, in vivo, and clinical setting. Therefore, our results highlight the clinically meaningful benefit and provide positive evidence to motivate further studies on improving long-term patient quality of life. Compared to two RCTs using first-in-class regimens^5,16^, two recent RCTs^17,18,22^, which utilized a second-generation BTKi (acalabrutinib) and/or novel third-generation anti-CD20 mAbs (obinutuzumab or ublituximab), have demonstrated superior outcomes of combination therapy versus BTKi alone. Acalabrutinib demonstrated better selectivity for BTK than ibrutinib^43^ and non-inferior survival outcomes with fewer cardiovascular AEs^44^. Next-generation anti-CD20 antibodies mediated superior antibody-dependent cellular cytotoxicity compared with rituximab in CLL^45,46^ and were more effective than rituximab among patients with CLL^8^. These findings can be helpful for immunologists and clinicians to establish further randomized studies by using next-generation anti-CD20 mAbs and next-generation BTKi.The main strength of the result derives from its large sample size and the pooled results. Two RCTs demonstrated improved PFS, CR rate, and MRD negativity rate, and the last two demonstrated no advantage compared to BTKi monotherapy. In comparison, the pooling of analysis from four RCTs allows us to see that the additional administration of anti-CD20 antibody significantly improved these outcomes compared to BTKi alone. Our study is the first systematic review and meta-analysis to demonstrate an improvement in efficacy and similar safety with the addition of anti-CD20 mAb to BTKi, which could affect treatment options for CLL. Another strength stems from the added value to elderly patients with relapsed or refractory disease. These patients are not considered eligible for high-dose chemotherapy and allogeneic stem cell transplantation because of comorbidities^47,48^ or are not practical due to the high cost of chimeric antigen receptor T-cell therapy due to qualitative T-cell defects in patients with CLL^49,50^. The median age of patients in all four RCTs was > 65 years. Therefore, the results can be applied to older patients and encourage trials on different combination targeted therapies in this population. Next, the median follow-up period was long enough to draw a reliable conclusion. Finally, our findings provide new evidence for therapy in patients with treatment-naïve or relapsed/refractory CLL. The results were consistently observed across high-risk genomic features. The efficacy of adding an anti-CD20 antibody to BTKi in treatment-naïve CLL patients had not been previously reported in an RCT. Indeed, this evidence provides the clinical potential of combining anti-CD20 antibody and BTKi regimen as first-line therapy to enhance clinical outcomes and potentially curative treatment of patients with CLL.

Several limitations of this study need to be considered. The main limitation is that the two novel treatments are ground-breaking^8,51,52^, and we have minimal RCTs. Another limitation stems from its variability in the methodology of the RCTs, leading to high heterogeneity between the trials. Although the studies have many similarities, there are still differences, including the procedure treatment and initial response assessment time. The inability to provide the same initial time assessment has considerable selection bias potential. Our study included two trials in CLL patients with relapsed/refractory and two trials in CLL patients with treatment-naïve, leading to a high degree of heterogeneity across the patients. In the two studies conducted in the relapsed/refractory setting, Sharman et al.^18^ did not report the number and related outcome of patients who were refractory to previous anti-CD20 therapy whereas Burger and colleagues did not mention the included patients who received the prior anti-CD20 treatment or not^16^. Although this limitation may have impacted results among relapsed/refractory patients, both intervention and control groups in the four included trials were generally well balanced, especially the previous line of therapy. Furthermore, in the ELEVATE-TN and GENUINE trials, only patients with complete response or partial response underwent central assessment of MRD while an assessment of MRD in bone marrow in most included patients was performed at cycle 9 in the ALLIANCE trial and at cycle 12 and 24 in the Burger et al. trial. A substantial test bias could arise from these trials. However, the MRD negative rates were calculated as the number of cases with negative MRD patients out of the total number of randomized patients in both arms according to the intention-to-treat population. It could minimize the impact of bias as a result. Given several limiting factors in this study, more well-designed randomized trials will soon be needed to detect any differences in overall survival.

## Conclusion

We have successfully demonstrated that the administration of add-on anti-CD20 mAb in BTKi has significantly superior outcomes compared to BTKi monotherapy. Despite the pooled analysis arising from only four pioneering RCTs and some considered limitations, future trials designed with next-generation BTKi and anti-CD20 antibodies need to improve these results and determine the optimal front-line strategy for managing treatment-naïve or relapsed/refractory patients with CLL.

## Supplementary Information

Supplementary Information.

## Data Availability

Data were extracted and analyzed from published articles, all available and accessible in the shared database. All datasets generated during the study are available upon reasonable request from the corresponding authors. The study protocol has been published (PROSPERO ID: CRD42022368514; www.crd.york.ac.uk/PROSPERO/) and is universally available.
