# Supplementary material for: Efficacy and safety of add-on anti-CD20 monoclonal antibody to Bruton tyrosine kinase inhibitor treatment for chronic lymphocytic leukemia: a meta-analysis
Source: Sci Rep. 2023 Jun 16;13:9775. doi: 10.1038/s41598-023-36279-x (PMC10276018; doi:10.1038/s41598-023-36279-x)

Efficacy and safety of add-on anti-CD20 monoclonal antibody to Bruton tyrosine kinase inhibitor treatment for chronic lymphocytic leukemia: A meta-analysis

Thi Thuy Nguyen[1,2], Nguyen Thanh Nhu [1,3], Van Khoi Tran[1,4], Nguyen-Kieu Viet-Nhi[1], Xuan Dung Ho[2], Ming-Kai Jhan [5,6], Ya-Ping Chen [7], and Chiou-Feng Lin [5,6,8,*]

SUPPLEMENTARY INFORMATION

Table S1. Search strategy (all fields included)

Table S2. Summary of adverse events associated with infusion-related reactions

Figure S1. Forest plot for subgroup analysis of progression-free survival according to del[17p] and/or mutated tumor protein 53 (a) status and del[11q] (b)

Figure S2. The pooled relative risk for subgroup complete response (a) and undetectable minimal residual disease rate (b) to treatment

Figure S3. The pooled risk ratio for discontinuation treatment rate (a) and risk ratio for death rate (b) to treatment

Table S1. Search strategy (all fields included)

**PubMed**		
**No**	**Query**	**Results**
#1	"chronic lymphocytic leukaemia"[All Fields] OR "leukemia, lymphocytic, chronic, b cell"[MeSH Terms] OR ("leukemia"[All Fields] AND "lymphocytic"[All Fields] AND "chronic"[All Fields] AND "b cell"[All Fields]) OR "b-cell chronic lymphocytic leukemia"[All Fields] OR ("chronic"[All Fields] AND "lymphocytic"[All Fields] AND "leukemia"[All Fields]) OR "chronic lymphocytic leukemia"[All Fields] OR "CLL"[All Fields]	30,771
#2	("anti-CD20"[All Fields] AND ("antibodies, monoclonal"[MeSH Terms] OR ("antibodies"[All Fields] AND "monoclonal"[All Fields]) OR "monoclonal antibodies"[All Fields] OR ("monoclonal"[All Fields] AND "antibody"[All Fields]) OR "monoclonal antibody"[All Fields])) OR ("rituximab"[MeSH Terms] OR "rituximab"[All Fields] OR "rituximab s"[All Fields] OR ("rituximab"[MeSH Terms] OR "rituximab"[All Fields] OR "rituxan"[All Fields] OR "rituximab s"[All Fields]) OR ("rituximab"[MeSH Terms] OR "rituximab"[All Fields] OR "mabthera"[All Fields])) OR ("obinutuzumab"[Supplementary Concept] OR "obinutuzumab"[All Fields] OR ("obinutuzumab"[Supplementary Concept] OR "obinutuzumab"[All Fields] OR "gazyva"[All Fields]) OR ("obinutuzumab"[Supplementary Concept] OR "obinutuzumab"[All Fields] OR "gazyvaro"[All Fields]) OR ("obinutuzumab"[Supplementary Concept] OR "obinutuzumab"[All Fields] OR "afutuzumab"[All Fields]) OR "GA101"[All Fields]) OR ("ublituximab"[Supplementary Concept] OR "ublituximab"[All Fields] OR ("ublituximab"[Supplementary Concept] OR "ublituximab"[All Fields] OR "tg 1101"[All Fields])) OR ("ofatumumab"[Supplementary Concept] OR "ofatumumab"[All Fields] OR ("ofatumumab"[Supplementary Concept] OR "ofatumumab"[All Fields] OR "arzerra"[All Fields]) OR ("ofatumumab"[Supplementary Concept] OR "ofatumumab"[All Fields] OR "kesimpta"[All Fields]) OR "HuMax-CD20"[All Fields])	30,713
#3	"ibrutinib"[Supplementary Concept] OR "ibrutinib"[All Fields] OR "ibrutinib s"[All Fields] OR ("ibrutinib"[Supplementary Concept] OR "ibrutinib"[All Fields] OR "imbruvica"[All Fields] OR "ibrutinib s"[All Fields]) OR ("ibrutinib"[Supplementary Concept] OR "ibrutinib"[All Fields] OR "pci 32765"[All Fields]) OR "CRA-032765"[All Fields] OR ("acalabrutinib"[Supplementary Concept] OR "acalabrutinib"[All Fields] OR ("acalabrutinib"[Supplementary Concept] OR "acalabrutinib"[All Fields] OR "calquence"[All Fields]) OR ("acalabrutinib"[Supplementary Concept] OR "acalabrutinib"[All Fields] OR "acp 196"[All Fields])) OR ("zanubrutinib"[Supplementary Concept] OR "zanubrutinib"[All Fields] OR ("zanubrutinib"[Supplementary Concept] OR "zanubrutinib"[All Fields] OR "brukinsa"[All Fields]) OR ("zanubrutinib"[Supplementary Concept] OR "zanubrutinib"[All Fields] OR "bgb 3111"[All Fields])) OR ("tirabrutinib"[Supplementary Concept] OR "tirabrutinib"[All Fields] OR "Velexbru"[All Fields] OR ("tirabrutinib"[Supplementary Concept] OR "tirabrutinib"[All Fields] OR "ono 4059"[All Fields]) OR ("tirabrutinib"[Supplementary Concept] OR "tirabrutinib"[All Fields] OR "gs 4059"[All Fields])) OR "Orelabrutinib"[All Fields] OR	6,821

	("Pirtobrutinib"[All Fields] OR "LOXO-305"[All Fields]) OR ("Nemtabrutinib"[All Fields] OR ("arq531"[Supplementary Concept] OR "arq531"[All Fields] OR "arq 531"[All Fields]) OR "MK-1026"[All Fields]) OR ("agammaglobulinaemia tyrosine kinase"[MeSH Terms] OR ("agammaglobulinaemia"[All Fields] AND "tyrosine"[All Fields] AND "kinase"[All Fields]) OR "agammaglobulinaemia tyrosine kinase"[All Fields] OR ("bruton"[All Fields] AND "tyrosine"[All Fields] AND "kinase"[All Fields]) OR "bruton tyrosine kinase"[All Fields] OR "BTK"[All Fields])	
#4	"randomized controlled trial"[Publication Type] OR "randomized controlled trials as topic"[MeSH Terms] OR "randomized controlled trial"[All Fields] OR "randomised controlled trial"[All Fields] OR "RCT"[All Fields]	781,915
#5	#1 AND #2 AND #3 AND #4	45
**EMBASE**		
#1	'chronic lymphatic leukemia'/exp/mj	30,805
#2	'cd20 antibody'/exp/mj	950
#3	'rituximab'/exp/mj	21,730
#4	'obinutuzumab'/exp/mj	1,064
#5	'ublituximab'/exp/mj	119
#6	'ofatumumab'/exp/mj	749
#7	#2 OR #3 OR #4 OR #5 OR #6	23,966
#8	'ibrutinib'/exp/mj	3,808
#9	'acalabrutinib'/exp/mj	482
#10	'zanubrutinib'/exp/mj	249
#11	'tirabrutinib'/exp/mj	72
#12	'orelabrutinib'/exp/mj	28
#13	'pirtobrutinib'/exp/mj	44
#14	'nemtabrutinib'/exp/mj	9
#15	'bruton tyrosine kinase'/exp/mj	969
#16	#8 OR #9 OR #10 OR #11 OR #12 OR #13 OR #14 OR #15	5,262
#17	'randomized controlled trial'/exp	735,831
#18	#1 AND #7 AND #16 AND #17	104
**MEDLINE**		
#1	chronic lymphocytic leukemia.mp. or Leukemia, Lymphocytic, Chronic, B-Cell/ OR CLL.mp.	28,300
#2	anti-CD20 monoclonal antibody.mp.	1,466
#3	Rituximab.mp. or Rituximab/ OR Rituxan.mp. or Rituximab/ OR MabThera.mp. or Rituximab/	29,248
#4	Obinutuzumab.mp. OR Gazyva.mp. OR Gazyvaro.mp. OR afutuzumab.mp.	802

	OR GA101.mp.	
#5	Ublituximab.mp. OR TG-1101.mp.	52
#6	Ofatumumab.mp. OR Arzerra.mp. OR Kesimpta.mp. OR HuMax-CD20.mp.	721
#7	#2 OR #3 OR #4 OR #5 OR #6	30,070
#8	ibrutinib.mp. OR Imbruvica.mp. OR PCI-32765.mp.	3,291
#9	acalabrutinib.mp. OR Calquence.mp. OR ACP-196.mp.	360
#10	zanubrutinib.mp. OR Brukinsa.mp. OR BGB-3111.mp.	199
#11	Tirabrutinib.mp. OR Velexbru.mp. OR ONO-4059.mp. OR GS-4059.mp.	89
#12	Orelabrutinib.mp.	13
#13	Pirtobrutinib.mp. OR LOXO-305.mp.	33
#14	Nemtabrutinib.mp. OR ARQ 531.mp.	10
#15	Bruton tyrosine kinase.mp. or Agammaglobulinaemia Tyrosine Kinase/ OR BTK.mp.	4,330
#16	#8 OR #9 OR #10 OR #11 OR #12 OR #13 OR #14 OR #15	6,371
#17	randomized controlled trial.mp.	618,369
#18	#1 AND #7 AND #16 AND #17	45
**Cochrane library**		
#1	chronic lymphocytic leukemia OR CLL	2,261
#2	anti-CD20 monoclonal antibody	426
#3	Rituximab OR Rituxan OR MabThera	5,634
#4	Obinutuzumab OR Gazyva OR Gazyvaro OR Afutuzumab OR GA101	467
#5	Ublituximab OR TG-1101	50
#6	Ofatumumab OR Arzerra OR Kesimpta OR HuMax-CD20	318
#7	#2 OR #3 OR #4 OR #5 OR #6	6,091
#8	Ibrutinib OR Imbruvica OR PCI-32765 OR CRA-032765	703
#9	Acalabrutinib OR Calquence OR ACP-196	158
#10	Zanubrutinib OR Brukinsa OR BGB-3111	71
#11	Tirabrutinib OR Velexbru OR ONO-4059 OR GS-4059	31
#12	Orelabrutinib	8
#13	Pirtobrutinib OR LOXO-305	20
#14	Nemtabrutinib OR ARQ 531 OR MK-1026	1
#15	Bruton tyrosine kinase	456
#16	#8 OR #9 OR #10 OR #11 OR #12 OR #13 OR #14 OR #15	999
#17	randomized controlled trial	949,304
#18	#1 AND #7 AND #16 AND #17	267

Total	461

Table S2. Summary of adverse events associated with infusion-related reactions

Infusion-related reaction	BTK inhibitor+anti-CD20			BTK inhibitor alone		
	Any grade	≥Grade 3	Total	Any grade	≥Grade 3	Total
Burger JA *et al.* 2019	5	0	104	0	0	104
Sharman JP *et al*. 2021	31	2	59	0	0	58
Sharman JP *et al*. 2020	25	5	178	0	0	179
Woyach JA *et al*. 2018	NR	0	181	0	0	180

Abbreviations: BTK, Bruton tyrosine kinase; NR, not reported.

Figure S1. Forest plot for subgroup analysis of progression-free survival according to 17p deletion and/or mutated tumor protein 53 (a) status and 11q deletion (b)

(a)

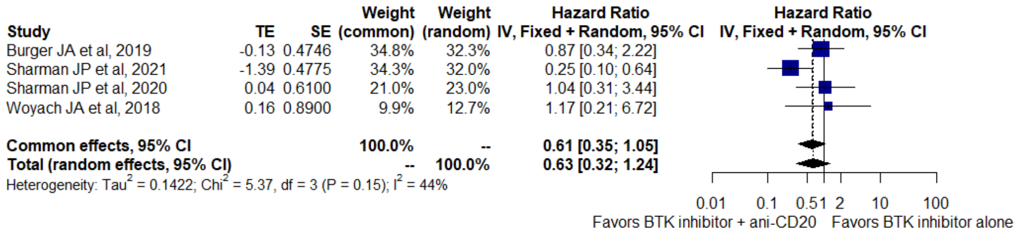

(b)

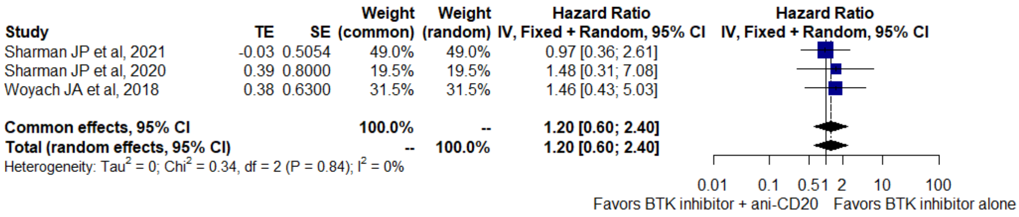

Abbreviations: BTK, Bruton tyrosine kinase; TP53, tumor protein 53; NR, not reported.

Figure S2. The pooled relative risk for subgroup complete response (a) and undetectable minimal residual disease rate (b) to treatment

(a)

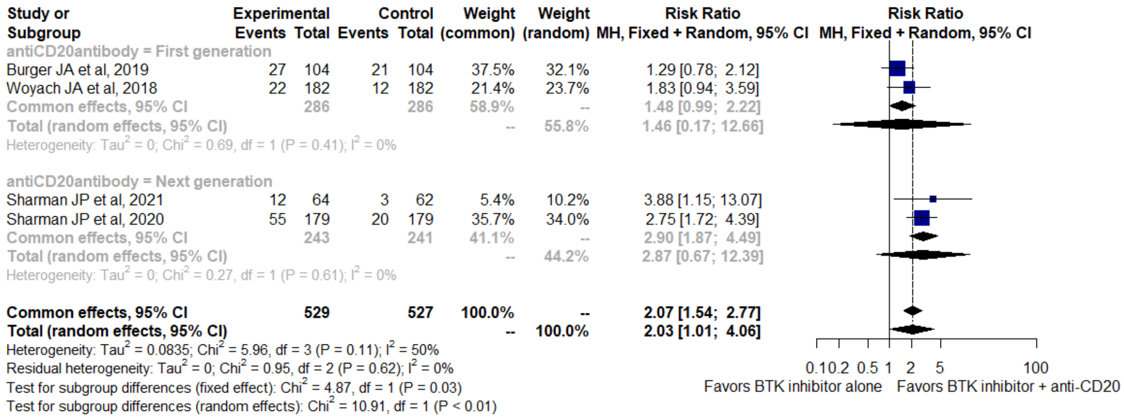

b)

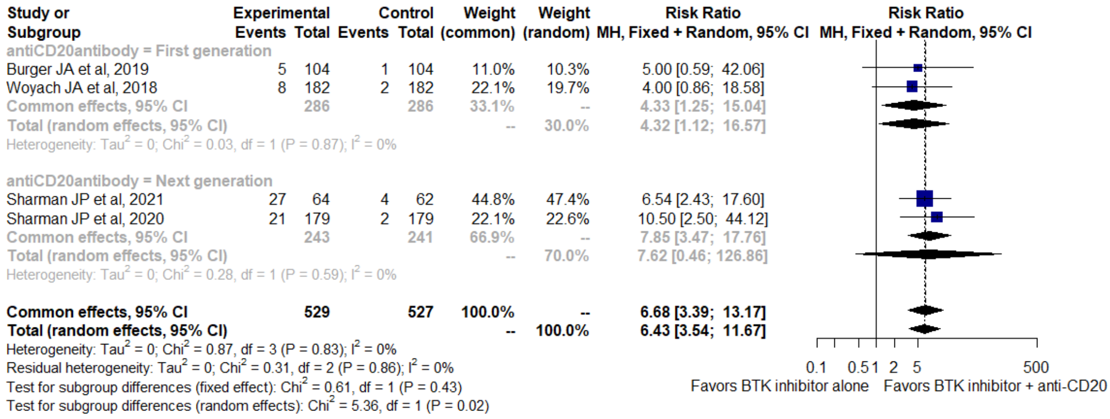

Figure S3. The pooled risk ratio for discontinuation treatment rate (a) and risk ratio for death rate (b) to treatment

(a)

Study	Experimental Events	Experimental Total	Control Events	Control Total	Weight (common)	Weight (random)	Risk Ratio MH, Fixed + Random, 95% CI
Burger JA et al, 2019	37	104	33	104	25.1%	27.3%	1.12 [0.76; 1.64]
Sharman JP et al, 2021	32	59	43	58	33.1%	40.2%	0.73 [0.55; 0.97]
Sharman JP et al, 2020	45	178	55	179	41.8%	32.5%	0.82 [0.59; 1.15]
**Common effects, 95% CI**		**341**		**341**	**100.0%**	**--**	**0.87 [0.71; 1.05]**
**Total (random effects, 95% CI)**					**--**	**100.0%**	**0.85 [0.50; 1.45]**

Heterogeneity: $Tau^2 = 0.0174$; $Chi^2 = 3.14$, df = 2 (P = 0.21); $I^2 = 36\%$

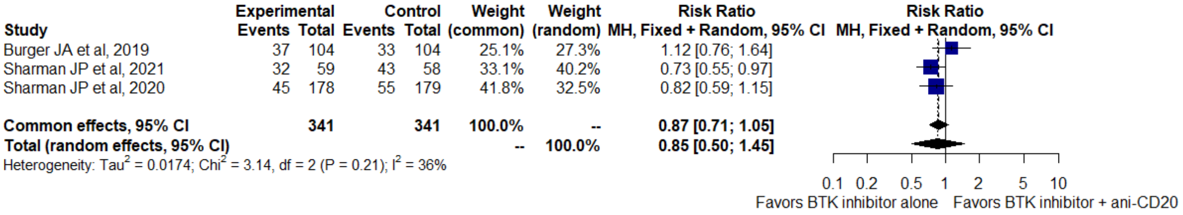

(b)

Study	Experimental Events	Experimental Total	Control Events	Control Total	Weight (common)	Weight (random)	Risk Ratio MH, Fixed + Random, 95% CI
Burger JA et al, 2019	9	104	7	104	11.8%	14.1%	1.29 [0.50; 3.32]
Sharman JP et al, 2021	10	59	16	58	27.3%	25.7%	0.61 [0.30; 1.24]
Sharman JP et al, 2020	8	178	12	179	20.2%	16.8%	0.67 [0.28; 1.60]
Woyach JA et al, 2018	22	181	24	180	40.7%	43.4%	0.91 [0.53; 1.57]
**Common effects, 95% CI**		**522**		**521**	**100.0%**	**--**	**0.83 [0.58; 1.18]**
**Total (random effects, 95% CI)**					**--**	**100.0%**	**0.82 [0.52; 1.30]**

Heterogeneity: $Tau^2 = 0$; $Chi^2 = 1.86$, df = 3 (P = 0.60); $I^2 = 0\%$

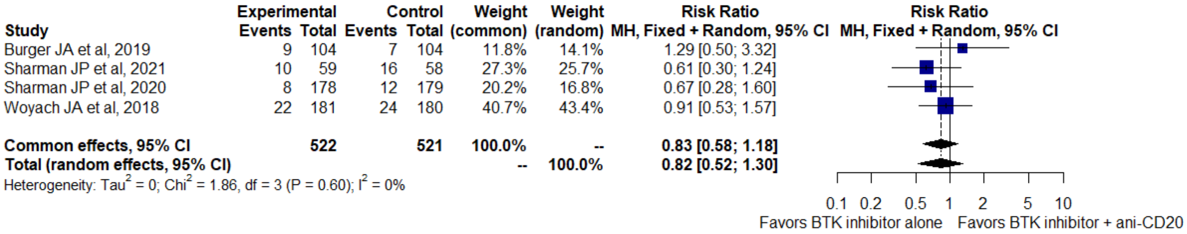

Supplement: Supplementary file 1 — Supplementary Information. [file 41598_2023_36279_MOESM1_ESM.pdf]
